# Assessment of Long-Term Effects of Sports-Related Concussions: Biological Mechanisms and Exosomal Biomarkers

**DOI:** 10.3389/fnins.2020.00761

**Published:** 2020-07-30

**Authors:** Aurélie Ledreux, Moira K. Pryhoda, Kim Gorgens, Kevin Shelburne, Anah Gilmore, Daniel A. Linseman, Holly Fleming, Lilia A. Koza, Julie Campbell, Adam Wolff, James P. Kelly, Martin Margittai, Bradley S. Davidson, Ann-Charlotte Granholm

**Affiliations:** ^1^Knoebel Institute for Healthy Aging, University of Denver, Denver, CO, United States; ^2^Department of Mechanical and Materials Engineering, University of Denver, Denver, CO, United States; ^3^Graduate School of Professional Psychology, University of Denver, Denver, CO, United States; ^4^Biological Sciences, University of Denver, Denver, CO, United States; ^5^Pioneer Health and Performance, University of Denver, Denver, CO, United States; ^6^Denver Neurological Clinic, Denver, CO, United States; ^7^Marcus Institute for Brain Health, Department of Neurology, University of Colorado Anschutz Medical Campus, Aurora, CO, United States; ^8^Department of Chemistry and Biochemistry, University of Denver, Denver, CO, United States

**Keywords:** concussion, mild traumatic brain injury, biomechanics, blood biomarkers, exosomes, neuropathology, neurodegenerative disease risk

## Abstract

Concussion or mild traumatic brain injury (mTBI) in athletes can cause persistent symptoms, known as post-concussion syndrome (PCS), and repeated injuries may increase the long-term risk for an athlete to develop neurodegenerative diseases such as chronic traumatic encephalopathy (CTE), and Alzheimer’s disease (AD). The Center for Disease Control estimates that up to 3.8 million sport-related mTBI are reported each year in the United States. Despite the magnitude of the phenomenon, there is a current lack of comprehensive prognostic indicators and research has shown that available monitoring tools are moderately sensitive to short-term concussion effects but less sensitive to long-term consequences. The overall aim of this review is to discuss novel, quantitative, and objective measurements that can predict long-term outcomes following repeated sports-related mTBIs. The specific objectives were (1) to provide an overview of the current clinical and biomechanical tools available to health practitioners to ensure recovery after mTBIs, (2) to synthesize potential biological mechanisms in animal models underlying the long-term adverse consequences of mTBIs, (3) to discuss the possible link between repeated mTBI and neurodegenerative diseases, and (4) to discuss the current knowledge about fluid biomarkers for mTBIs with a focus on novel exosomal biomarkers. The conclusions from this review are that current post-concussion clinical tests are not sufficiently sensitive to injury and do not accurately quantify post-concussion alterations associated with repeated mTBIs. In the current review, it is proposed that current practices should be amended to include a repeated symptom inventory, a cognitive assessment of executive function and impulse control, an instrumented assessment of balance, vestibulo-ocular assessments, and an improved panel of blood or exosome biomarkers.

## Introduction

Up to 3.8 million sports-related concussions (mild traumatic brain injury; hereafter referred to as mTBI) are diagnosed annually in the United States; however, it is estimated that only 50% of mTBIs are reported (Broglio et al., 2017; Iverson et al., 2017). A potential consequence of mTBI is post-concussion syndrome (PCS), defined as the occurrence of clinical symptoms that continue for weeks, or months following the incident (Sosnoff et al., 2011; Berz et al., 2013; Tagge et al., 2018). Related PCS physical symptoms can include headache, dizziness, insomnia, exercise intolerance, fatigue, as well as noise, and light sensitivity (Ling et al., 2015; Cottle et al., 2017; Iverson et al., 2017; Gozt et al., 2020). Psychological symptoms including depression, irritability, and anxiety are common. Cognitive problems such as memory loss, poor concentration, and reduced problem-solving skills can also persist over time (McIinnes et al., 2017; Howell et al., 2018a). These long-lasting physical, psychological, and cognitive symptoms can significantly affect quality of life, prevent return to work and add a financial burden on individuals experiencing PCS (Voormolen et al., 2019). Studies have identified a number of risk factors including age, sex, learning issues, history of migraine that could influence development of PCS (Iverson et al., 2017). Both acute and chronic PCS have been identified in the literature, each presenting with unique health concerns (Tagge et al., 2018; Yin et al., 2019; Scott et al., 2020). In addition, there is increased concern that repeated mTBIs may contribute to adverse long-term consequences including neurodegenerative diseases (Asken et al., 2016; McAllister and McCrea, 2017). It is thought that one or several mTBIs give rise to alterations in inflammatory processes in the brain via activation of astrocytes and microglial cells (Mouzon et al., 2014; Papa et al., 2015; Winston et al., 2016), as well as the more direct neuronal insults including axonal degeneration, neuronal cell loss (Daneshvar et al., 2015; McKee and Daneshvar, 2015), and intracranial accumulation of dementia-related damaging proteins including amyloid beta peptide (Aβ), and phosphorylated forms of Tau (p-Tau; Papa et al., 2015; Gill et al., 2018). In a recent study, our group demonstrated that neuron-derived exosomal (NDE) biomarkers, isolated from blood samples, exhibit significant alterations both short- and long-term following one or repeated mTBIs (Goetzl et al., 2019a). Although research on the short- and long-term effects of mTBI have been conducted in animal models (see below), reliable and reproducible assessments for clinical use including biomarkers are still under development in this field. The development of biomarker panels is important since they may predict or quantify long-term health risks for adverse outcomes and may represent a more objective measure of the effectiveness of interventions. Development of novel assessment tools is needed to better quantify injury in the short-term and to fully understand the long-term consequences of mTBIs for brain health.

Demographic studies have identified several risk factors for sustaining sports-related mTBIs. The greatest risk of sustaining an mTBI exists among males playing American football or Australian rugby, females playing soccer (Giza et al., 2013; Kutcher and Giza, 2014), and both genders playing ice hockey (Simmons et al., 2017). Athletes participating in lacrosse were recently shown to have a high risk for mTBIs, although fewer studies have been conducted in terms of injury consequences in lacrosse compared to other high impact sports (Reynolds et al., 2016; Miyashita et al., 2017; O’connor et al., 2017). Athletes playing baseball, softball, volleyball, and gymnastics reportedly have a lower risk for sustaining mTBIs compared to other contact sports (Giza et al., 2013). Across all sports, females, and younger players were reported to have an increased risk of developing PCS after sustaining one or repeated mTBIs (Giza et al., 2013; Collins et al., 2014; Kutcher and Giza, 2014). A personal history of migraine, depression, previous concussions, learning disabilities, or attention-deficit hyperactivity disorder (ADHD) also increases the risk for developing PCS following mTBI (Giza et al., 2013; Bramley et al., 2016). Thus, several different factors impact the risk for long-term consequences after mTBI, including the severity of the injury, the type of sports and position played, age of the injured person (younger individuals suffer fewer long-term consequences), number of previous mTBIs, and the addition of repeated subclinical, “sub-concussive” injuries (Asken et al., 2016). The demographics of our cohort of National Collegiate Athletic Association (NCAA) Division 1 athletes resembles the demographics described above, in terms of increased risks sustained in athletes playing ice hockey, or lacrosse (see Table 1). An increase in concussions in skiers (44% with a history of concussions), similar to previously reported findings (Gil et al., 2017) was also observed.

**TABLE 1:** History of concussion in cohort by sport and gender.

**Sport**	**Total number of athletes**	**Number of athletes with history of concussion**	**Percent of athletes with history of concussion**	**Male/Female with history of concussion**
Basketball	34	14	41%	8/6
Diving	6	3	50%	2/1
Golf	10	0	0%	0/0
Gymnastics	15	4	27%	0/4
Hockey	43	21	49%	21/0
Lacrosse	116	46	40%	31/15
Skiing	18	8	44%	3/5
Soccer	48	21	44%	9/12
Swimming	27	4	15%	3/1
Tennis	20	3	15%	3/0
Volleyball	16	4	25%	0/4
Total	353	128	36%	80/48

Research on the typical neurocognitive sequelae of concussions suggests there is little uniformity in the presentation and course of PCS (Leddy et al., 2016). Despite that, the diagnosis of concussion is commonly based on physical, cognitive, and emotional symptoms, including the presence or absence of loss of consciousness and/or amnesia, behavioral changes, and/or sleep disturbance (Kutcher and Giza, 2014; Broglio et al., 2017; Cottle et al., 2017; Iverson et al., 2017). The NCAA adopted its Concussion Policy and Legislation in 2010 (Baugh et al., 2015), which recommends that all athletes participate in a brain injury/concussion history survey, symptom evaluation, cognitive assessment, and balance evaluation prior to participation in a collegiate sport but these measures of cognitive change, balance, and self-reported distress are only moderately sensitive to mTBI (Higgins et al., 2017). Further, despite these guidelines, many competitive athletes underreport post-concussive symptoms and fail to report concussive injuries (Covassin et al., 2007; Kroshus et al., 2015; McDonald et al., 2016; Wallace et al., 2017). As a result, they do not receive the clinical attention needed to promote recovery and avoid short- and long-term risks.

The accurate assessment of mTBI and post-concussive symptoms is crucial to athlete safety and is often at the center of return-to-play (RTP) decisions for healthcare providers and sports medicine staff (Baugh et al., 2015; Broglio et al., 2017). Importantly, premature cognitive and physical activity prior to the recovery of concussive injury is associated with prolonged neuronal vulnerability, often increasing the severity of and delaying the resolution of PCS (Harmon et al., 2013). Additionally, individuals who sustain one or several additional concussions may be more vulnerable to a worsening of post-concussive neurological and metabolic dysfunction and potentially to long-term harm to the brain (Prins et al., 2010; Kenney et al., 2018). Despite the increased attention to PCS and the risk of long-term negative effects on brain health, few quantitative methods are available to fully assess these risks. There is an urgent need to improve TBI diagnosis and support recovery in the short-term and to understand and reduce the long-term risk of adverse outcomes including neurodegenerative conditions such as chronic traumatic encephalopathy (CTE) or Alzheimer’s disease (AD) that are often associated with repeated concussive events.

The objective of this review is to provide an overview of: (1) clinical and biomechanical tools available to sport medicine practitioners to ensure recovery after mTBIs before RTP, (2) potential biological mechanisms in animal models underlying long-term adverse consequences of mTBIs, (3) the link between repeated mTBI and neurodegenerative diseases, and (4) current knowledge of fluid biomarkers for mTBIs, with an emphasis on blood biomarkers and on emerging exosomal biomarkers. More research into specific mechanisms of injury is needed to improve the prognostics for sport-related mTBIs and a better understanding of the neuronal consequences reflected by plasma or exosome biomarkers is also necessary.

## Current Assessment Tools for mTBI

General guidance for the management of sports-related mTBIs includes an assessment of symptoms and disposition after first addressing acute trauma (McCrory et al., 2017). Injury management guidelines and many state statutes require that concussed players not be allowed to return to play on the day of the injury (Harmon et al., 2013; McCrory et al., 2017). A graduated six-step RTP protocol after a concussion is suggested by the Consensus Statement on Concussion in Sport (McCrory et al., 2017; Patricios et al., 2018).

The diagnosis of concussion has historically been based on self-reported symptoms (Kasamatsu et al., 2016) including nausea, dizziness, or headache. Six core symptom scales have been developed for sport-related concussion (Alla et al., 2011; Thomas et al., 2011) and many of them are variants of the original Pittsburgh Steelers Post-Concussion Scale (Alla et al., 2011; McLeod and Leach, 2012). These scales were developed from clinical experience and consensus rather than empirical evidence (Cantu, 1997; Leclerc et al., 2001; Tagge et al., 2018).

The Concussion in Sport Group (CISG) first introduced the Sport Concussion Assessment Tool (SCAT) 1 to provide a multifaceted standardized assessment of concussion which has since evolved into the SCAT2, SCAT3, and SCAT5 (McLeod and Leach, 2012; Yengo-Kahn et al., 2016; Echemendia et al., 2017; Joseph et al., 2018). The diagnostic utility of the SCAT appears to diminish 3–5 days post-injury (Yengo-Kahn et al., 2016), making this test less effective for evaluating post-acute effects including PCS. Additionally, consensus statements from the CISG state that grading systems should not be used to dictate concussion management, and that the SCAT5 symptom checklist does not demonstrate clinical utility in tracking recovery (McCrory et al., 2017). Despite that, 87% of athletic trainers report that they will return a non-symptomatic athlete to play based only on self-reported symptoms (Covassin et al., 2009). This may be especially problematic in collegiate or professional sports where athletes have a financial incentive to play. For those reasons, self-reported symptom scores should always be used in tandem with objectively measured domains during concussion management. Given the heterogeneity of mTBI presentation and the high stakes of athlete health, there is an ongoing need to develop more sensitive instruments and individualized injury management protocols (Choe and Giza, 2015).

Because cognitive dysfunction has been demonstrated to take longer to resolve than the self-reported symptoms (Carman et al., 2015), computerized neurocognitive tests have become the most commonly used objective clinical measure of mTBI. The Immediate Post-Concussion Assessment and Cognitive Test (ImPACT; Kontos et al., 2014; Higgins et al., 2017) is widely used in both youth and NCAA sports for concussion management in the United States. ImPACT was found to have 81.9% sensitivity and 89.4% specificity to mTBI (Schatz et al., 2006) and generates composite scores for four domains: Verbal Memory, Visual Memory, Processing Speed, and Reaction Time, of which visual memory, and reaction time are the most sensitive domains to the cognitive changes following concussion (Covassin et al., 2007; Majerske et al., 2008). Other computerized neurocognitive assessment tools such as the ANAM^TM^ Core Battery (Kane et al., 2007) can measure changes in attention, working memory, and cognitive efficiency, and may therefore be even more sensitive to the long-term effects of mTBI on brain health (Covassin et al., 2007; Kane et al., 2007; Majerske et al., 2008; Kontos et al., 2014; Martini et al., 2017). Preliminary data from our cohort (Gorgens et al., unpublished observations) strongly suggest that executive function, reaction time, and processing speed should be included in the routine assessment of PCS, since these cognitive changes appear to linger after most other cognitive functions have returned to baseline.

Vestibular- and ocular-based clinical tests are also sensitive to concussive injury. The vestibular and ocular systems contribute critical information on spatial orientation to the central nervous system (CNS) and detriments in the vestibulo-ocular system can lead to dizziness, nausea, and balance impairment. In a battery of vestibular-based laboratory tests, post-concussion deficits can be identified using the Dynamic Visual Acuity Test (DVAT; Zhou and Brodsky, 2015), and the King-Devick (KD) test (King et al., 2015). The KD test measures timed saccadic eye movements and has been shown to have 86% sensitivity and 90% specificity to concussion (Galetta et al., 2011), and is also sensitive to unreported mTBIs (King et al., 2015). The addition of the KD test may increase the overall sensitivity of a clinical concussion battery.

The call for more sensitive concussion assessment and the growing awareness of CNS compensatory function has increased the utility of balance testing. Compensatory CNS function is observed when, for example, an “eyes closed” balance task results in instability. In that case, vision can be said to compensate for deficits in the vestibular system (Sosnoff et al., 2011). In fact, the visual system has been determined by sensory organization testing to be dominant over the vestibular system in balance tasks (Liaw et al., 2009; Allum and Honegger, 2016). An mTBI results in a deficit of vestibular function and so, when balancing, concussed patients will compensate for this loss using the visual system.

The Balance Error Scoring System (BESS; Finnoff et al., 2009; Yengo-Kahn et al., 2016; Miyashita et al., 2017) is an observational diagnostic tool frequently used to test static balance post-mTBI on firm and foam surfaces with the eyes closed. The BESS is the most commonly used balance metric for sideline concussion diagnosis despite concerns that it does not adequately assess vestibular function (Kalajainen, 2015) and issues about learning effects with repeated testing (Valovich et al., 2004; Mulligan et al., 2013). However, the BESS is used in most NCAA protocols for mTBIs, as this assessment is readily available in the field. Instrumented measurements of static balance have been found to be less subject to scoring errors than observational balance tools and more sensitive to mTBI-related changes in balance. For example, concussed football players showed greater center of pressure displacement immediately after injury with an improvement of function before RTP (Powers et al., 2014).

Measuring dynamic balance (balance during movement) may be a better indicator of athlete readiness to RTP. However, clinicians are currently limited in their ability to measure dynamic balance as those methods are still being optimized. A slower normal gait speed (Howell et al., 2018a) has been documented among patients with mTBIs. Tandem gait, defined as walking toe to heel, is also slower for the first three days after concussion, and dual task tandem gait time is slower for the first two weeks post-mTBI (Howell et al., 2018a). Other measurements such as smaller step width (Buckley et al., 2019) and slower dual task cadence (Howell et al., 2018b) have been documented post-mTBI. Gait disturbance has also been found in neurodegenerative disease and aging, which may give insight into longer-term effects of multiple mTBIs. For example, the walking speed of patients with AD is slower than healthy older adults (Johansson et al., 2017) and walking speed differences appear already in those with mild cognitive impairment (MCI; Knapstad et al., 2019), suggesting that gait change is an early event and can be used to diagnose potential problems. It is therefore possible that long-term tracking of gait post-mTBI can reveal future risk for developing neurodegenerative conditions related to multiple mTBIs and may represent a sensitive dynamic balance measure for these cases.

Chen and Chou (2010) have created a clinically-accessible dynamic biomechanical balance measure using reflective marker data from the ankle to create a Center of Mass (COM)-ankle inclination measure. COM medio-lateral displacement is typical of concussed patients in the first two days post-concussion (Grants et al., 2017) and up to 2 months post-concussion (Howell et al., 2018a). Peak anterior COM velocity can also be measured and is observed to be greater for the first two months post-concussion (Howell et al., 2018a). There is continued work on the development of a commercially-viable quantitative measurement of dynamic balance. The use of the Kinect system (Microsoft, United States) for spatiotemporal parameters of gait has been promising (Zhu et al., 2017).

In sum, there is a growing base of concussion research that confirms our understanding of concussion as a multi-faceted injury, affecting both cognitive, and balance systems. To date, the measurement of mTBI-related balance and cognitive sequelae as required by NCAA regulations is moderately sensitive to short-term concussion effects, but less sensitive for long-term PCS.

## Potential Biological Mechanisms – Animal Studies

Most biological mechanisms for long-term effects of mTBIs have been discovered via animal models. There have been several thorough reviews published previously describing the relative pros and cons of various mouse and rat models of TBI (Lyeth, 2016; Osier and Dixon, 2016; Wojnarowicz et al., 2017). Therefore, only a brief overview is provided here. There are many distinct methods for inducing TBI in rodent models for research purposes. A partial listing of the many variants of these models is provided by Xiong et al. (2017a). However, in terms of mTBI or concussion, it could be argued that *the most physiologically relevant models* are those that utilize pneumatically-driven or electromagnetic impact devices to deliver single or repetitive closed head injuries to mice or rats. Recent studies have begun to use un-anesthetized animals and animals in which the head is free to move in response to the impact rather than being immobilized. These models may ultimately prove to replicate the dynamic injury of mTBI more accurately than those using anesthetized and immobilized animals; however, they are not without some level of controversy.

### Diffuse Axonal Injury

Several models of repetitive mTBIs have demonstrated persistent multi-focal axonal injury using electron microscopy, silver staining, and diffusion tensor imaging (Shitaka et al., 2011; Bennett et al., 2012). Axonal injury was prevalent in repetitive mTBI models 3 to 6 months post-injury (Ojo et al., 2016). Moreover, in comparison to the axonal injury observed following a single mTBI, repetitive mTBIs exacerbated axonal degeneration and this degenerative process was ongoing in subcortical white matter tracts for up to 24 months post-injury, suggesting long-term damage (Laurer et al., 2001; Mouzon et al., 2012, 2014, 2018). Thus, repetitive mTBIs can induce chronic and progressive axonal degeneration which undoubtedly contributes to cognitive and perhaps emotional deficits post-mTBI. It seems likely that this ongoing degenerative process may set the stage for the development of chronic neurodegenerative disorders, even though definitive proof of this link has not yet been provided in humans.

### Tau Pathology

Although neither of the specific Tau pathologies of AD or CTE have been precisely replicated in mouse models of mTBI, marked alterations in Tau phosphorylation, and processing do occur in mice following TBIs. For instance, a single unilateral impact in un-anesthetized mice induced rapid tauopathy within 24 h of injury, which persisted and progressed to the contralateral uninjured cortex by 5.5 months post-injury (Tagge et al., 2018). Several other models of single or repetitive mTBIs also produce aberrant Tau pathology (Kane et al., 2012; Luo et al., 2014; Petraglia et al., 2014; Yang et al., 2015; Ignowski et al., 2018). Of note, un-anesthetized mice administered 6 concussive impacts daily for 7 days, displayed elevated cortical p-Tau immunoreactivity up to 6 months post-mTBI (Petraglia et al., 2014). Collectively, these results demonstrate that increased p-Tau is observed in diverse mouse models of mTBI. Moreover, this phospho-Tau pathology often persists for several months post-injury and has the potential to “spread” to areas of the brain which are outside of the primary injury site via a seeding/prion-like mechanism, perhaps via exosomes (Deleo and Ikezu, 2018; Winston et al., 2019a). Therefore, these mouse studies have been essential for informing the biomarker field as to which biomarkers of disease may be of the most relevance to long-term pathology in the brain.

The effects of repetitive mTBIs have been investigated in two mouse models of tauopathy. In transgenic mice expressing the shortest human Tau isoform (hTau23), repetitive mTBI induced extensive telencephalic neurofibrillary tangles (NFTs) in only one of twelve transgenic mice (Yoshiyama et al., 2005). In a second model, aged (18-month-old) mice, which express multiple wild-type human Tau isoforms (hTau mice), were subjected to either a single or repeated mTBIs. Although these aged hTau mice already express significant Tau pathology, only repetitive mTBI markedly increased hyper-phosphorylation of Tau as assessed by multiple phospho-Tau specific antibodies. Thus, it appears that repetitive mTBIs have the potential to exacerbate certain forms of Tau pathology, although precipitation of *bona fide* NFTs is not efficiently induced by this trauma/gene interaction paradigm, at least not in mouse models. To our knowledge, only one laboratory has described a mouse model which seems to meet (most of) these criteria. This model induces a tauopathy marked by hyperphosphorylation which was detectable in cortical axons ipsilateral to a cortical impact injury by 24 h post-injury and in neuronal soma by 2 weeks post-injury (Tagge et al., 2018). Moreover, the Tau pathology “spread” to the distant cortex in a bilateral fashion at 5.5 months post-injury. Finally, these authors have also demonstrated that *cis* phospho-Tau is a proximal driver of neuronal injury post-TBI and that treatment with a specific *cis* phospho-Tau blocking antibody significantly attenuates Tau pathology and spread (Albayram et al., 2017), providing potential new avenues for development of effective preventative methods after repeated mTBIs in humans.

### Neuroinflammation

**FIGURE 1:**
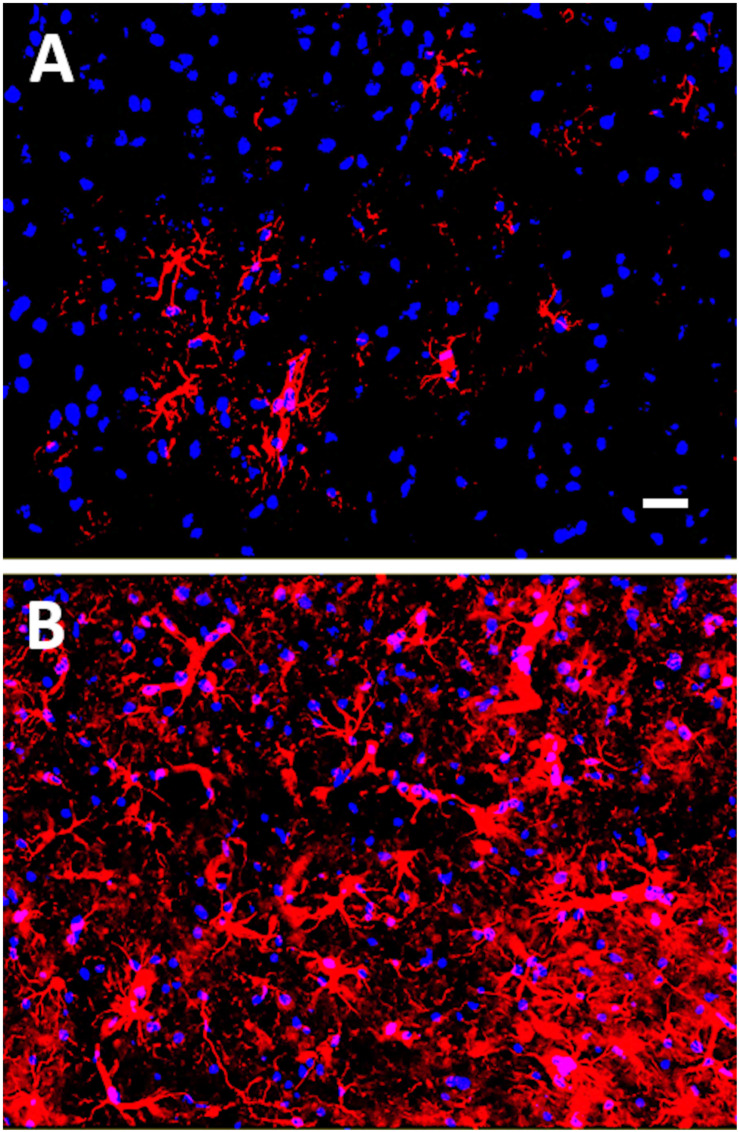
Glial fibrillary acidic protein (GFAP) immunostaining of mouse cortex 18 days post-mTBI induced by a closed head injury using an electromagnetically controlled impact device. In **(A)**, cortex of a Sham mouse, and in **(B)**, cortex of a mouse subjected to mTBI. GFAP-positive astrocytes are shown in red (Cy3) and nuclei are shown in blue (Hoechst dye). Note the increased gliosis in mouse cortex following the mTBI. Scale bar represents 20 microns.

It is well known that recovery from neurotrauma, such as a stroke or TBI, can be confounded by the brain’s ability to perpetually activate microglia. Reactive gliosis following neural injury can lead to chronic inflammation and neurodegeneration (Turner et al., 2016). Undoubtedly, neuroinflammation is the most common pathological finding in diverse mouse models of mTBI and is an undeniable contributing factor in chronic neurodegenerative disease states (Mouzon et al., 2014; Daneshvar et al., 2015; McAteer et al., 2016; Winston et al., 2016; Edwards et al., 2017). The activation of astrocytes in response to neuronal damage, i.e., astrogliosis, has been identified as an early event following a single mTBI in juvenile mice subjected to closed head injury, adult mice exposed to controlled cortical impact, and adult un-anesthetized mice given a lateral closed head impact injury (Susarla et al., 2014; Israel et al., 2016; Chen et al., 2017; Rodriguez-Grande et al., 2018; Tagge et al., 2018). In some cases, astrogliosis persisted for up to 28 days post-TBI, which was the longest time point analyzed post-injury in these studies (Susarla et al., 2014). Our group has similarly observed astrogliosis in mice exposed to mTBI induced by controlled cortical impact (see Figure 1; Ignowski et al., 2018). In mice subjected to repeated mTBIs, long-term cognitive deficits were associated with persistent astrogliosis up to one-year post-injury (Mannix et al., 2013). Crawford and colleagues have examined the lifelong consequences of repetitive mTBIs in mice and found that neuroinflammation progressed over 12 to 24 months, appearing in subcortical white matter tracts in association with degenerating axons at these later time points post-injury (Mouzon et al., 2012, 2014, 2018). These findings suggest that repetitive mTBIs can precipitate a lifelong neurodegenerative process. Neuroinflammation has been observed as a contributing factor, both in CTE (Daneshvar et al., 2015; McAteer et al., 2016; Tagge et al., 2018), and in AD (Heneka et al., 2015).

### Alzheimer’s Disease (AD)

In two distinct models of Aβ precursor protein (APP) mutagenesis, the Tg2576 mouse and the APP/PS1 transgenic mouse, mTBI enhanced Aβ aggregation, and precipitated cognitive impairment of pre-symptomatic AD mice (Uryu et al., 2002; Tajiri et al., 2013). In contrast, repetitive mTBI did not increase Aβ or phospho-Tau levels in the 3xTg-AD mouse model. However, axonal degeneration and microglial activation were observed in these animals up to 1 year post-TBI (Winston et al., 2016). Thus, consistent findings from several groups unequivocally suggest that neurotrauma does at the very least accelerate the development of AD changes in genetically predisposed rodents. Overall, these findings are somewhat variable, but they suggest that mTBI administered on a genetic background of human genes expressing AD pathology tends to accelerate amyloid pathology, hippocampal neurodegeneration, and neuroinflammation. Additional studies are underway in our group to further investigate the effects of mTBI on the progression of AD neuropathology.

In conclusion, these previous studies suggest that a genetic propensity for AD pathology is exacerbated by repeated mTBIs, thus providing a potential biological mechanism for the fact that not everyone with multiple mTBIs, either resulting from active duty or from high impact sports, succumb to neurodegenerative diseases including CTE when they get older. Animal studies are valuable in that they can dissect long-term effects in a relatively short time period, and researchers can modify specific genetic factors to explore their impact on long-term brain health.

## Concussion and Neurodegenerative Diseases

Although several sources conclude no connection between exposure to a single mTBI and incidence of neurodegenerative disease (Guskiewicz et al., 2005; Carson, 2017; Manley et al., 2017; McCrory et al., 2017; Lobue et al., 2020), other studies suggest that repeated mTBIs, without full recovery time between events, could increase the risk for neurodegenerative disease decades later (Guskiewicz et al., 2005; Cruz-Haces et al., 2017). Repetitive injuries are known to increase the risk for future injury and are associated with slower recovery after an mTBI including the persistence of PCS (Daneshvar et al., 2011). Guskiewicz et al. (2005) documented earlier than typical onset of AD in a cohort of retired American football players. In that study, the football players who reported three or more mTBIs had five times greater MCI diagnoses and three times greater reported memory problems compared to players who did not report a history of mTBIs. CTE is a complex neurological disorder characterized by executive dysfunction, depression, memory impairment, and dementia, amongst other types of cognitive and affective dysfunctions (Daneshvar et al., 2015; Asken et al., 2016; McKee et al., 2016; Armstrong et al., 2017). CTE has been strongly associated with one major TBI or repeated milder mTBIs.

In a recent *post mortem* study, investigators found evidence of CTE in the brains of teenage athletes following repeated mTBIs (Tagge et al., 2018), suggesting that the onset of pathology may be earlier than previously thought. The neuropathological alterations that occur following repeated brain injuries include diffuse axonal damage (Lepage et al., 2018), white matter degeneration, widespread neuroinflammation with activated microglial cells, and astrocytes (Chen et al., 2017; Collins-Praino and Corrigan, 2017; Johnson et al., 2017), lipid peroxidation and other oxidative damage (Yates et al., 2017), as well as accumulation of intracellular NFTs caused by p-Tau (Olczak et al., 2017). The accumulation of NFTs has been observed in the deep sulci in frontal and temporal cortices and along cortical blood vessels of individuals with CTE and is thought to contribute significantly to the long-term development of neurodegeneration in individuals with mTBIs (Omalu, 2014; McKee et al., 2016; Washington et al., 2016; Mez et al., 2017; Alosco et al., 2018). Dizziness and imbalance, both common symptoms of mTBI, have been linked to white matter abnormalities and diffuse axonal injuries (Cecil et al., 1998), in addition to vestibular system damage (Akin et al., 2017). Diffuse axonal injuries following mTBIs have also been reported in experimental animal models of TBI (Mannix et al., 2013).

Tau pathology induced by mTBI may be related to both AD and CTE, which each have unique presentations of p-Tau species. In AD, Tau is hyper-phosphorylated and aggregated into NFTs that are found throughout the hippocampus and cortex and exhibit a progressive spread eventually reaching all cortical areas (Raj et al., 2012; Kaufman et al., 2016; Furman et al., 2017). In contrast, the Tau pathology in CTE is characterized by the accumulation of abnormal (and hyperphosphorylated) Tau focally around small blood vessels at the depths of the sulci within the cortex, essentially at the white matter-gray matter interface (Omalu, 2014; McKee et al., 2016; Mez et al., 2017; Olczak et al., 2017; Tagge et al., 2018). Notably, Tau filaments in AD and CTE assume different molecular structures (Fitzpatrick et al., 2017; Falcon et al., 2019). In a recent study by Mez et al. (2017), the investigators examined the brains of 202 former American football players who had passed away. CTE was diagnosed in 177 of these players, with the mildest pathology observed in athletes who had only played football in high school, and the most severe pathology in players who had a professional football career (Mez et al., 2017). Eighty-six percent of the athletes who played professionally exhibited severe CTE-related neuropathology. Among the participants with severe CTE pathology, 85% showed clinical signs of dementia. Even though this was a select collection of brain tissue obtained because the athletes actively donated their brains to this particular project, these findings are alarming and suggest that sustaining repeated mTBIs is related to significant detriments later in life. These pathology studies also suggest that total Tau and p-Tau need to be included in any blood biomarker panel that is used to assess long-term mTBI effects, and that repeated mTBIs earlier in life most certainly predisposes individuals to neuropathology later in life.

Mild traumatic brain injurys may also trigger other neurodegenerative diseases including Parkinson’s disease (PD) and amyotrophic lateral sclerosis (ALS; Gupta and Sen, 2016; Gardner et al., 2018), although there are less quantifiable data available to firmly connect mTBIs with the incidence of either PD or ALS to date. The strong connection between p-Tau and CTE as well as AD has prompted a wealth of investigations to reveal blood or cerebrospinal fluid (CSF) biomarkers related to toxic forms of Tau both acutely and chronically after mTBIs. TBI and PCS share many clinical symptoms with CTE and may even precede the development of CTE. Although most biomarker studies to date have been focused on short-term (weeks or months) consequences of mTBIs, one should also take into consideration the long-term effects (years or decades) of repeated mTBIs that take place in youth or collegiate high-impact sports.

## Biomarkers in mTBI

### Blood Biomarkers

The relationship between multiple mTBIs and neurodegenerative diseases is a controversial issue that is currently being debated in several lawsuits. Because of the inherent difficulty in providing tangible evidence of long-term risk after multiple mTBIs in professional players, prognostic biomarkers are needed to provide confirmation of damage and association with neurodegenerative conditions. Fluid biomarker research has gained increasing attention over the last decade for its promise to help quantify injury severity, determine when it is safe for athletes to return to play, and to track the long-term consequences of mTBIs for brain health. That research also highlights the underlying biological mechanisms of mTBI and can be used to evaluate the effectiveness of therapeutic interventions (O’connell et al., 2018). However, plasma and serum biomarkers are variable in athletes who have sustained mTBIs and may not truly reflect simultaneous alterations occurring in the brain. Moreover, recent data suggest that the glymphatic clearance system [i.e., the macroscopic waste clearance system utilizing perivascular channels to eliminate toxins and soluble proteins from the brain’s interstitial space while distributing glucose, lipids and amino acids to support brain’s functioning (Jessen et al., 2015)] is impaired in the acute phase of mTBI, showing a 60% decreased clearance efficiency for up to 1 month post-mTBI (Iliff et al., 2014). Although supporting evidence from human cohorts is currently lacking, it appears that glymphatic dysfunction following mTBI may result in low blood biomarker levels because of their compromised clearance (Plog and Nedergaard, 2015).

In addition, current biomarkers are not necessarily adequate to examine the long-term risk associated with mTBIs. While other potential biomarkers exist such as Positron Emission Tomography (PET), Magnetic Resonance Imaging (MRI) modalities, and computerized electroencephalogram (EEG), this review focuses solely on fluid biomarkers including exosomes. Existing fluid biomarkers have often shown inconsistent results across published studies. A number of fluid biomarkers have been examined for their ability to assess the severity of mTBI (Zetterberg et al., 2013; Papa, 2014; Papa et al., 2015; Kulbe and Geddes, 2016) but a panel consisting of multiple biomarkers may have greater sensitivity and specificity and therefore increase diagnostic accuracy (Yokobori et al., 2013; McCrea et al., 2020). Several studies suggest that exosome biomarkers may provide a consistent and sensitive assessment of ongoing brain-health issues (Fiandaca et al., 2015; Kapogiannis et al., 2019; Goetzl et al., 2019a). Therefore, we propose the use of exosomal biomarkers for assessment of PCS in athletes. This approach will also be beneficial for the much larger populations of veterans and civilians who sustain mTBIs outside of sports.

To date, there are essentially three classes of fluid biomarkers that can be used to identify mTBI: Biomarkers associated with (1) neuronal/axonal damage, (2) activation of glial cells, and (3) release of inflammatory cytokines that occur due to neuronal injury. Neuronal biomarkers that have been examined include neuron-specific enolase (NSE), calpain-derived *N*-terminal fragment of αII spectrin (SNTF), Tau, neurofilament-light (NF-light), ubiquitin C-terminal hydrolase-L1 (UCH-L1), and brain-derived neurotrophic factor (BDNF). Glial activation markers include S100 calcium binding protein B (S100B) and glial fibrillary acidic protein (GFAP). Lastly, inflammatory cytokines have been measured both in CSF and blood and include the pro-inflammatory cytokines interleukins-1, -12, and -18 (IL-1, -12, and -18), tumor necrosis factor alpha (TNF-α), and interferon gamma (IFN-γ). Over the last few years, much attention has been brought to the development of blood biomarkers that can accurately diagnose mTBI acutely or predict the long-term changes in the brain associated with one or repeated mTBIs (Zetterberg et al., 2013; Kulbe and Geddes, 2016), and early studies have identified some promising candidates.

#### Neuronal Biomarkers

The first neuron-specific biomarker to be explored for mTBI was NSE (Stalnacke et al., 2004). NSE is involved in axonal transport and elevations of NSE following an injury initiates an inflammatory cascade (Kawata et al., 2016; Haque et al., 2018). Graham and colleagues showed that amateur boxers experiencing direct punches to the head had increased serum NSE levels compared to those who took punches to the body (Graham et al., 2011). Interestingly, following a 2-month resting period, serum levels of NSE remained significantly elevated in boxers compared to healthy non-boxer controls, suggesting that repetitive head trauma leads to prolonged neuronal damage (Zetterberg et al., 2009). However, a recent meta-analysis of 10 independent studies showed that NSE levels were not a strong independent predictor of PCS following mTBI (Mercier et al., 2018). Further, NSE is sensitive to hemolysis and is also present in erythrocytes and endocrine cells, and could be a potential confounder (Ramont et al., 2005).

**SNTF (calpain-cleaved α-spectrin N-terminal fragment)** has been identified as a protein that also accumulates preferentially in damaged axons, as a result of neuronal stretch injury (Johnson et al., 2016). In a study of concussed ice hockey players, Siman and colleagues reported that serum SNTF levels were increased 1 h following an mTBI and remained significantly elevated from 12 h to 6 days, before declining to pre-season baseline levels (Siman et al., 2015). SNTF is not a commonly used biomarker but might prove valuable once it has been more thoroughly investigated. In a recent meta-analysis, Gan et al. (2019) found that SNTF was a top candidate to predict delayed recovery after mTBI. **Neurofilament-light (NF-light)** is another biomarker for axonal damage that can be measured in serum. NF-light levels are increased in ice hockey players with PCS (Shahim et al., 2017) and in active professional fighters as well as in retired fighters with neurological issues (Bernick et al., 2018). However, no difference in serum NF-light levels were found between baseline and 6-day and 14-day post mTBI in a cohort of adolescent athletes with uncomplicated mTBI, suggesting that the severity of the neuronal injury might have been too low to detect changes in NF-light levels (Wallace et al., 2018).

**Ubiquitin carboxy-terminal hydrolase L1 (UCH-L1)** is an enzyme involved both in the processing of ubiquitin precursors and of ubiquitinated proteins and is highly expressed in the neuronal cytoplasm (Setsuie and Wada, 2007). Serum UCH-L1 has a high accuracy albeit with a high risk of bias in predicting cat scan (CT) findings in mTBI and appears as a promising blood-based diagnostic biomarker for TBI, but further studies are needed (Shahjouei et al., 2018). UCH-L1 interacts with both p-Tau and Aβ, making the ubiquitin pathway a promising target for development of novel treatment avenues for neurodegenerative disease (Papa et al., 2015; Joseph et al., 2018; McCrea et al., 2020). McCrea and colleagues (McCrea et al., 2020) recently showed that serum UCH-L1 levels were acutely elevated in concussed athletes compared to baseline preseason levels but that levels had returned to baseline 48 h after injury or 7 days after RTP. It was recently reported significant elevations in UCH-L1 levels in neuron-derived exosomes (NDEs) obtained from athletes with acute, but not chronic, mTBIs suggesting that this marker may be an excellent predictor of short-term effects (Goetzl et al., 2019a).

Gill et al. (2017) showed that, in collegiate athletes, increased plasma **total Tau** concentration within 6 h of mTBI correlated to having an extended RTP duration. In a recent study of collegiate football players, the investigators found significant elevations in plasma Tau levels post-practice, but no correlation between the number of sub-concussions or mTBIs and Tau (Kawata et al., 2016). Alosco et al. (2018) found that greater exposure to repetitive head impacts in a cohort of 96 former NFL players correlated with higher plasma total Tau concentrations later in life. Pathological phosphorylation of Tau has been associated with long-term risk for neurodegenerative disease, such as CTE after repeated mTBIs (Baugh et al., 2015; Daneshvar et al., 2015; Asken et al., 2016; Di Battista et al., 2016; Broglio et al., 2017; Alosco et al., 2018; Barnes et al., 2018; Goetzl et al., 2019b). **p-Tau** is considered an important biomarker for determining long-term effects of mTBIs in terms of risk for developing dementias because neuropathological findings confirm that p-Tau accumulates in the brain within NFTs in both AD and in CTE (Tracy and Gan, 2018). Our recent findings indicate that levels of p-Tau (T181 and S396) exhibit long-term changes when measured in NDE preps from blood (Goetzl et al., 2019a), strongly suggesting that this novel method for assessing biomarkers in exosomes of neuronal origin may provide an important insight into brain-specific events after injury. Although these are highly experimental procedures to date, they hold promise for development of successful targeting of Tau pathology in the near future.

**Brain-derived neurotrophic factor (BDNF)** is a neurotrophic factor highly involved in brain health, with serum levels increasing after physical exercise or cognitive training (Hakansson et al., 2017; Ledreux et al., 2019). It has been suggested that BDNF-related treatments could offer neuroprotection and help facilitate neuroplastic changes to reverse deficits associated with TBI (Kaplan et al., 2010). Korley et al. (2016) have found decreased day-of-injury BDNF levels after TBI compared to non-TBI controls.

#### Glial Activation Biomarkers

Astroglial proteins such as **S100 calcium-binding protein B (S100B)** and **glial fibrillary acidic protein (GFAP)** are proteins located in the cytoplasm of astrocytes and have received a lot of attention as markers of glial activation, particularly following mTBIs (Kawata et al., 2016; Sajja et al., 2016; Bogoslovsky et al., 2017). The levels of S100B can help differentiate patients with mTBI from those with severe TBI and was shown to improve predictions of long-term post-concussion outcomes (Dey et al., 2017). However, there is evidence that S100B is not only produced in the brain but also in peripheral cells such as adipocytes and chondrocytes (Diaz-Romero et al., 2014), thus making its measurement in peripheral blood less specific to sport-related mTBI and providing a rationale for focusing on other glial biomarkers which could measure more directly effects of mTBIs associated with brain damage.

Neselius et al. (2012) showed that CSF levels of GFAP were increased within 6 days after an mTBI in more than 80% of a cohort of 30 Olympic boxers and returned to levels comparable to the control cohort after a rest period of 14 days. Interestingly, there are conflicting reports regarding the efficiency of GFAP as a biomarker in terms of predictive and diagnostic value for post-mTBI assessments. While several research groups found that GFAP was the most promising biomarker in terms of serum elevations after mTBI (Kou et al., 2013), others did not see significant increases in GFAP, even in athletes with mTBIs (Meier et al., 2017). Others have shown, using single-molecule array (Simoa) technology, that individuals with TBI exhibit elevated levels of GFAP immediately after arrival in the hospital (Bogoslovsky et al., 2017). In a recent study, McCrea and colleagues showed that athletes with mTBI had significantly elevated levels of GFAP compared to pre-season baseline levels, and that a more severe mTBI with loss of consciousness resulted in further elevated GFAP levels, thus suggesting that this biomarker can be of potential clinical utility (McCrea et al., 2020). Both glial markers GFAP and S100B are still among the most investigated biomarkers for mTBI, especially for short-term effects and for diagnostics in the emergency room following an injury. However, one should keep in mind the potential contribution of the glymphatic clearance system since preclinical data obtained in a murine TBI model demonstrated that inhibition of the glymphatic clearance (Jessen et al., 2015) suppressed TBI-induced increased serum levels of S100B and GFAP (Plog et al., 2015).

#### Inflammatory Cytokines

Release of inflammatory cytokines reflects the activation of glial cells in the brain, as well as increased activation of the peripheral immune system which can also indirectly affect neuroinflammation (Patterson and Holahan, 2012). mTBI is associated with neuroinflammation, expressed as elevated pro- and anti-inflammatory cytokines in both CSF and in blood (Hobbs et al., 2016; Singh et al., 2016). Neuroinflammation, which is a natural response to widespread injury in the brain, can be detrimental for neuronal survival and increases the activation of microglia and astrocytes in the vicinity of the injury. The severity of this secondary wave of injury, caused by inflammation and activation of glial cells, is highly influential on long-term outcomes (e.g., Jassam et al., 2017). In the subacute period after sport-related mTBI, Di Battista and colleagues found increased levels of MCP-4 and MIP-1β that could discriminate between athletes with mTBI compared to healthy controls in the acute phase post-injury, however, no significant differences were observed at the time of medical clearance (Di Battista et al., 2016). These authors also found that subacute levels of MCP-1 and MCP-4 were positively correlated with days to recovery in athletes with mTBI. In another study, symptom duration and IL-6 levels 6 h post-mTBI were significantly associated in high school and collegiate football players (Nitta et al., 2019), thus highlighting the clinical significance of inflammatory markers, at least for the acute phase after mTBI. While a limited number of studies have investigated the changes in inflammatory markers after mTBI, examination of inflammatory markers in neuron-, and astrocyte-derived exosomes (ADEs) could further our understanding of the neurophysiological consequences of sport-related mTBI since neuroinflammation appears to be a key player in neurodegenerative processes.

### Exosomal Biomarkers

As described above, blood levels of NF-light, GFAP, UCH-L1, Tau/p-Tau, and/or inflammatory biomarkers are promising for identifying the acute phase of mTBI but it has been shown that blood biomarkers have limitations for quantifying the chronic effects of repeated mTBIs (Agoston et al., 2017). On the contrary, CSF biomarkers can better quantify post-acute effects and inform the time line for RTP (Papa et al., 2015) but both animal and human studies of mTBI demonstrate the importance of serial sampling after a concussive event, which makes it difficult to rely on CSF biomarkers in clinical situations. Therefore, the identification of exosomal biomarkers involved in post-acute PCS could contribute significantly to the identification, diagnosis, and prognosis of the underlying individual pathobiological changes of mTBI and the long-term effects of repeated mTBIs.

Exosomes are nanosized extracellular vesicles that are produced by every cell type in the body and are released for degradation of cargo and/or signaling/transportation from cell to cell (Hamlett et al., 2019). By measuring total Tau levels in total exosomes from plasma, Stern and collaborators were able to discern between CTE and control patients more than 80% of the time. Interestingly, higher levels of exosomal tau were associated with worse performance on memory tests, but not with mood or behavior deficits (Stern et al., 2016). This correlation was not present when measuring total Tau in plasma, although this measure was positively associated with cumulative head impact index (Alosco et al., 2018). Guedes et al. (2020) measured plasma and exosomal levels of NF-light in veterans with history of mTBIs and found that those experiencing chronic PCS and post-traumatic stress disorder (PTSD) exhibited the highest NF-light levels both in plasma and exosomes.

The analysis of CNS-derived exosomes by ultra-high sensitivity detection methods is a novel method that has the potential to provide ongoing information regarding brain health both in athletes and service members exposed to one or several mTBIs. To our knowledge, Gill et al. (2018) were the first to demonstrate the effects of mTBIs on exosomal biomarkers. They utilized ultrasensitive single molecule array (Simoa) technology to measure Tau, Aβ42, and IL-10 levels in NDE from 42 military personnel with mTBI compared to 22 military with no mTBI. They found that NDE Tau levels were elevated following mTBIs compared to those with no mTBI (Gill et al., 2018). Further, they observed that within the mTBI group, post-concussive symptoms were most related to NDE Tau elevations, whereas NDE IL-10 levels were related to PTSD symptoms. This study strongly suggested that NDE biomarkers may be more sensitive and may also better reflect post-acute changes in the brain post-concussion than blood biomarkers. However, more experiments are needed to determine whether total Tau or p-Tau levels in CNS-derived exosomes can predict long-term conversion to CTE or other tauopathies in individuals with repeated mTBIs.

In a recent study by our group (Goetzl et al., 2019a), differential exosomal increases of certain proteins acutely following an mTBI (annexin VII, UCH-L1, claudin-5, aquaporin 4, and synaptogyrin-3), and chronically in student athletes with chronic mTBIs [>12 months prior; Aβ42, p-tau T181, p-tau S396, IL-6, and cellular prion protein (PRPc)] suggested a shift in pathological processes as a result of time after injury. Although these findings are promising, more studies are needed that examine exosomal cargo changes long-term after repeated mTBIs, as would be the case for student athletes playing high-impact sports.

Other content of exosomes, particularly their miRNA cargo, can impact the post-transcriptional regulation of many genes. miRNAs are small non-coding, single-stranded RNA molecules comprising of around 22 nucleotides (Zhang et al., 2015). miRNA carried by exosomes are involved in various key processes such as nerve and vascular regeneration and can either promote regeneration or aggravate degeneration (Kalani et al., 2014). Although miRNAs function in the brain is still being investigated, altered miRNA levels in blood and saliva following TBI have been reported, and several candidates miRNA have been identified as potential biomarkers for TBI (Atif and Hicks, 2019). Recently, using a combination of microchip diagnostic and machine learning, Ko et al. (2018) have developed a platform to isolate brain-derived circulating exosomes and measure multiple miRNAs packaged within these exosomes to accurately identify TBI patients from healthy controls. New treatment paradigms involving miRNA are also being explored and would be especially promising if it can be proven that a single miRNA has the ability to influence several target genes, making it possible for the researchers to potentially modify a whole disease phenotype by modulating a single miRNA molecule.

### Decreased Exosome Release After TBI

Concentrations of NDEs, assessed by counts and levels of exosome marker CD81, were significantly depressed by a mean of 45% after acute mTBI in collegiate athletes, but not in student athletes with chronic mTBI, compared with controls (Goetzl et al., 2019a). A recent manuscript also showed that the levels of ADEs were lower immediately after a severe TBI, but levels of ADEs returned to normal by 12 months post-TBI (Goetzl et al., 2020). On the other hand, Winston et al. (2019b) found no differences in CD81 levels between veterans with or without history of mTBI, highlighting the need for more research to examine the potential effects of timing of blood sampling after mTBI and source of exosomes as factors that could affect the exosome release. Others have shown that microvesicles released in the CSF from people with TBI had a smaller diameter than in controls (Manek et al., 2018).

**FIGURE 2:**
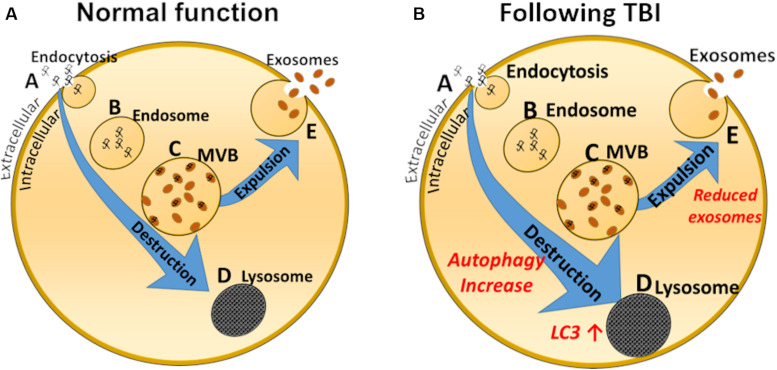
Schematic representation of exosome biology in a normal cell **(A)** and after mTBI **(B)**. Normally, endocytosis **(A)** leads to inclusions of early endosomes **(B)**, which in turn leads to development of multivesicular bodies (MVB, **C**), which can either be demolished by the lysosomal pathway **(D)**, or expelled from the cell as exosomes **(E)**. Studies suggest that after a TBI the lysosomal/autophagosomal pathway is increased, to the detriment of exosomal secretion. Autophagosomal markers, such as LC3 are elevated.

Mechanisms involved in these observations may be related to increased autophagy occurring after mTBIs or TBIs, as demonstrated by Smith et al. (2011) and illustrated by the schematic drawing depicted in Figure 2. A primary reason may be the increase in autophagy reported acutely after brain and spinal injury (Smith et al., 2011), perhaps leading to reduced exosomal production by default (Figure 2). Smith and collaborators propose that reduced exosomal release could be a physiological response, recycling injured organelles and other products of deterioration instead of releasing them into multivesicular bodies and exosomes. Relative levels of the autophagy marker LC3-II were increased by 100–200% in injured rat brains compared with sham rats (Liu et al., 2008), supporting this notion. LC3 immunoreactivity is also increased several-fold in rats after controlled cortical impact compared with sham injury, peaking at 8 days (Zhang et al., 2008). Other studies (Chen et al., 2018) suggest that inducing autophagy (for example by using Omega-3 supplements) might be beneficial for recovery after mTBIs, suggesting that elevated autophagy is a repair mechanism in the brain. The alteration in the exosomal/autophagy pathway after TBI or mTBI is diametrically different to that occurring for example in people with Down syndrome, where the levels of autophagy are reduced and levels of the exosomal marker CD63 are increased manifested by increased exosomal release (Hamlett et al., 2019). Of course, there are many pathological events that occur, both short- and long-term, after mTBIs that contribute to secondary damage, poor outcome and future increased risk for neurodegenerative conditions. For example, future research should focus on addressing how the blood-brain barrier integrity and glymphatic system dysfunction that have been described after TBI affect exosome production and release (Sullan et al., 2018; O’keeffe et al., 2019). Because regulation of autophagy can exhibit anti-oxidative stress, anti-apoptosis and anti-inflammatory effects following repeated mTBIs, this represents a promising target for further therapeutic development (Zhang and Wang, 2018). In addition, many neuroprotective drugs may attenuate TBI-induced secondary brain injury via activation of autophagy (Ding et al., 2015; Gao et al., 2017; Zhang et al., 2017).

## Potential Use of Exosomes for Treatment of TBI

**FIGURE 3:**
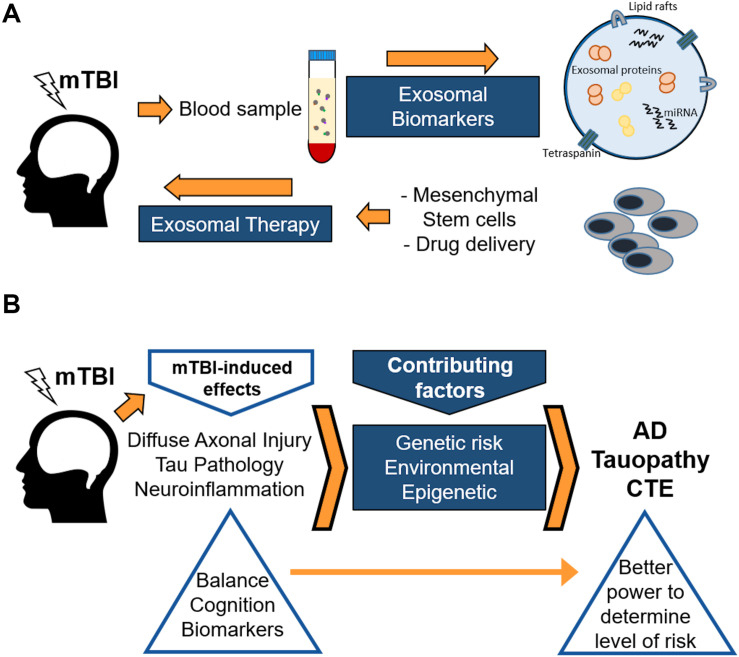
Schematic showing the use of exosomes both as biomarkers and therapeutic agents **(A)**, and a proposed protocol for genetic, epigenetic, and environmental factors involved in post-concussion outcomes in humans **(B)**. **(A)** Purifying either ADEs or NDEs from blood samples can lead to identification of brain-related factors, both proteins and miRNAs, that are involved in long-term or short-term post-concussion brain alterations. In addition, recent work from the cancer field suggests that exosomes obtained from enriched stem cells, such as mesenchymal stem cells, can be delivered in blood to reach and benefit brain areas affected by TBI. **(B)** Mild traumatic brain injury (mTBI) induces several adverse pathological effects in the brain. These effects, along with genetic, environmental, and epigenetic factors, contribute to an enhanced risk of neurodegeneration later in life. Neurodegeneration, as a result of the damage produced by mTBI, may later present itself in the form of Alzheimer’s disease (AD), tauopathy, chronic traumatic encephalopathy (CTE), and other maladies. Genetic, epigenetic, as well as environmental factors may affect the outcome after mTBIs. However, it may be possible to identify those individuals at increased risk of neurodegeneration due to mTBI by incorporating a more rigorous assessment using enhanced balance and cognitive testing along with biomarker studies following mTBI.

Novel data from several research groups strongly suggest that exosomes may serve not only as a biomarker source, but also as a delivery tool of novel treatment therapies for TBIs and mTBIs (Figure 3A). Xiong et al. (2017b) posed that exosomes are more stable and can cross the blood-brain barrier, compared with their parent cells. When administering, for example, mesenchymal stem cells (MSCs), there are major safety risks associated with the risk of occlusion in microvasculature or unregulated growth of transplanted cells in brain parenchyma (Xiong et al., 2017b). Therefore, the development of cell-free exosome-based therapy may provide a novel approach to enhancing neuroplasticity and amplifying neurological recovery following one or several mTBIs (see Figure 3A). Beneficial effects of exosomes derived from stem cells in rodent models for TBI have been demonstrated (Yang et al., 2017; Zhang et al., 2019). A huge advantage with these stem cell-derived exosomes is that they could be delivered systemically, reducing the need for more invasive treatment avenues. Li et al. (2017) suggested that the beneficial effects afforded by stem cell-derived exosomes on TBI recovery in a rat model were at least partially due to a shift in microglial phenotype from the M1 to the M2 phenotype, strongly indicating that inflammation plays a major role for long-term outcomes after TBIs. Finally, Kaijzel et al. (2017) suggest that nanoparticles, including exosomes and other cell-free delivery systems, may provide a more targeted approach for successful intervention and prevention therapies, to prevent long-term brain damage following one or several mTBIs. However, this promising field is yet in its infancy, although the cancer field has reached much further in the search for exosome-based therapies (Weston et al., 2019). Most likely, the TBI field can benefit from the findings in the cancer field and spur the development of promising new therapies.

## Summary and Conclusion

A single or repeated mTBI can cause short-term symptoms (collectively called PCS) and may also increase the risk for long-term neurodegenerative conditions including CTE, AD, or other neurodegenerative or neuropsychiatric conditions. Ongoing research suggests that current RTP protocols should evolve to include quantitative assessments of reported symptoms, cognition, vestibulo-ocular function, dynamic balance, and exosomal biomarkers. The expansion of that battery will confer a direct benefit to the athletic community and increase the safety of RTP protocols. It will also contribute to concussion management education and awareness as well as earlier detection of symptoms that may be related to long-term neurodegenerative disease.

Several studies have suggested that a battery including self-reported symptoms as well as a neurocognitive and balance tasks have a sensitivity to concussion exceeding 90%. However, most studies have not considered long-term PCS changes in the brain that may impact brain health. Therefore, it appears important to obtain and bank baseline data on all athletes. That includes individualized testing for balance, cognitive performance, and biomarkers. Our experience from a cohort consisting of more than 300 NCAA Division I athletes in high-impact sports clearly shows a need for expanded testing paradigms including instrumented balance measurements and better cognitive batteries that capture long-term changes in executive function, impulse control, and decision making, for example supplementing the ImPACT battery with portions of the ANAM test battery.

Although more research is needed to determine the most sensitive and specific biomarkers for long-term brain health, it seems appropriate to include a panel of biomarkers in mTBI protocols in cases where PCS appears (Gan et al., 2019; McCrea et al., 2020). Further, our data presented herein strongly suggest that exosomal biomarkers may represent a more sensitive and reliable biomarker method, at least for long-term effects. Our recent findings indicate that Tau, p-Tau S396, p-Tau T181, and Aβ42 exhibit long-term changes when measured in NDE preps from blood (Goetzl et al., 2019a), strongly suggesting that this novel method for assessing biomarkers in exosomes of neuronal origin may provide an important insight into brain-specific events after injury. Based on our findings and those by others, it is proposed herein that exosomes can be used both as a valuable “liquid biopsy” to determine what goes on in the brain – either glial cells or neurons – and can also be developed to a novel way to treat mTBIs, for example using exosomes derived from MSCs (Mondello et al., 2018; Figure 3). Animal and *in vitro* studies can reveal biological mechanisms involved in disease progress long-term, as discussed above. Based on previous and current findings in the field, a model is emerging in which diffuse axonal injury, Tau pathology, and neuroinflammation incrementally contribute to long-term changes in the brain that eventually lead to increased risk for tauopathies such as AD or CTE with aging.

## Author Contributions

A-CG, AL, DL, KG, and BD contributed to the conception of the review, coordinated writing efforts, and edited final article version. A-CG, AL, and KG wrote the Introduction. MP and BD wrote the balance assessment part. KG prepared the cognitive assessment part. AL and A-CG wrote the biomarker and exosome portions. DL, LK, and HF worked on the animal studies section. All authors collaborated with or were part of the DU Concussion research group and contributed to critical revisions of the manuscript, read, and approved the submitted version.

## Conflict of Interest

The authors declare that the research was conducted in the absence of any commercial or financial relationships that could be construed as a potential conflict of interest.
